# Estimation of Instantaneous Gas Exchange in Flow-Through Respirometry Systems: A Modern Revision of Bartholomew's Z-Transform Method

**DOI:** 10.1371/journal.pone.0139508

**Published:** 2015-10-14

**Authors:** Hodjat Pendar, John J. Socha

**Affiliations:** Department of Biomedical Engineering and Mechanics, Virginia Tech, Blacksburg, VA, 24061, United States of America; James Cook University, AUSTRALIA

## Abstract

Flow-through respirometry systems provide accurate measurement of gas exchange over long periods of time. However, these systems have limitations in tracking rapid changes. When an animal infuses a metabolic gas into the respirometry chamber in a short burst, diffusion and airflow in the chamber gradually alter the original signal before it arrives at the gas analyzer. For single or multiple bursts, the recorded signal is smeared or mixed, which may result in dramatically altered recordings compared to the emitted signal. Recovering the original metabolic signal is a difficult task because of the inherent ill conditioning problem. Here, we present two new methods to recover the fast dynamics of metabolic patterns from recorded data. We first re-derive the equations of the well-known Z-transform method (ZT method) to show the source of imprecision in this method. Then, we develop a new model of analysis for respirometry systems based on the experimentally determined impulse response, which is the response of the system to a very short unit input. As a result, we present a major modification of the ZT method (dubbed the ‘EZT method’) by using a new model for the impulse response, enhancing its precision to recover the true metabolic signals. The second method, the generalized Z-transform (GZT) method, was then developed by generalizing the EZT method; it can be applied to any flow-through respirometry system with any arbitrary impulse response. Experiments verified that the accuracy of recovering the true metabolic signals is significantly improved by the new methods. These new methods can be used more broadly for input estimation in variety of physiological systems.

## Introduction

**Fig 1:**
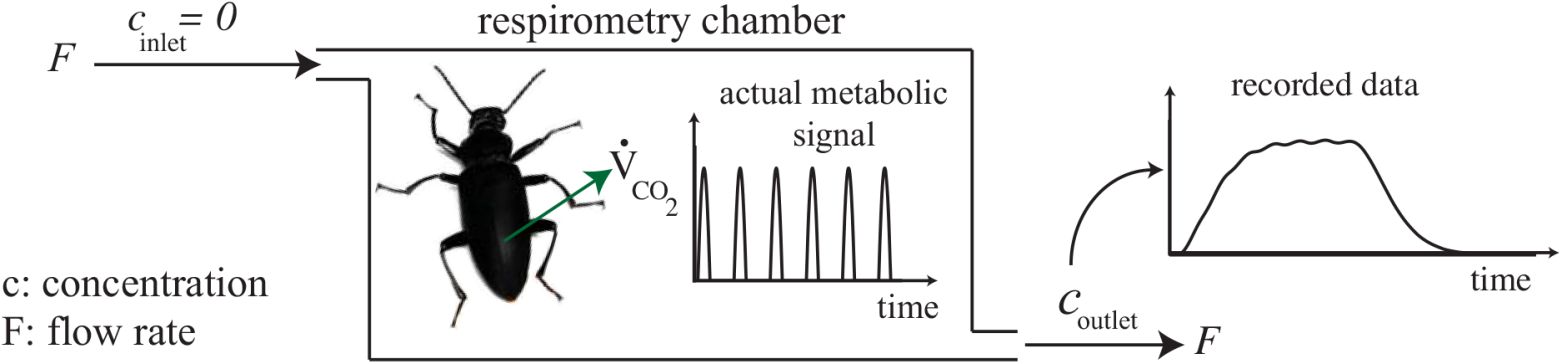
Washout problem in flow through respirometry systems. When the animal changes the concentration of a metabolic gas inside the respirometry chamber with a certain shape, it appears in a different pattern in the outlet.

Flow-through respirometry is an important and powerful tool for understanding physiology, providing a mechanism to estimate the metabolic rate of animals, plants, microorganisms, cells, and tissues with a broad range of sizes [1]. In this technique, the driven air through a respirometry chamber constantly replenishes the oxygen (O_2_) consumed by the animal while transporting carbon dioxide (CO_2_) and water vapor produced by the animal to a gas analyzer [1–4]. For animals, metabolic rate is estimated by measuring the oxygen consumption rate ${\dot{V}}_{O_{2}}$ and/or carbon dioxide production rate ${\dot{V}}_{CO_{2}}$ [5]. Despite a long tradition of using these measurements to calculate time-varying metabolic rates, it is well known that flow-through respirometry systems do not in fact measure gaseous signals instantaneously. This problem not only reduces the precision of measurement, it can also obscure important physiological information about the animal of study. The root of the problem results from volumetric washout of the gas of interest (O_2_, CO_2_, or water vapor) within the chamber, pipes, and the detection chamber of the gas analyzer. In practice, if the animal emits a short burst of CO_2_, the inlet airflow gradually washes the emitted CO_2_ molecules from the chamber and delivers them to the gas analyzer, changing the shape of the emitted burst (Fig 1). Depending on the chamber size and the flow rate, it can take several seconds to several hours to wash out all of the CO_2_ molecules from the single burst. During this period, the animal may produce other CO_2_ bursts that can intermix before entering the gas analyzer. The net effect of this process is alteration and/or superposition of the signals, resulting in recorded data that are different from the true gas exchange signal emitted from the animal.

In addition to changing the signal itself, another major problem with flow-through respirometry is synching gas exchange data with other physiological measurements. Processes of interest can range broadly from movement cycles during locomotion [6, 7] to food consumption in animals [8, 9]. The faster or more transient the process, the more important it is to understand the temporal dynamics of the true respiratory signal. For example, all insects periodically open and close their spiracles (valve-like structures that regulate respiration), and some species also collapse and re-inflate their tracheal tubes on the second to sub-second timescale [10–12]; the exact timing and pattern of these events governs the animal’s external gas exchange patterns. In studying this system, an improperly matched respiratory signal may lead an investigator to attribute peaks in CO_2_ release to spiracular opening rather than bulk flow created by tube collapse, or vice versa, leading to an incorrect interpretation of the insect’s respiratory physiology. Therefore, any interpretation of the dynamics of respiration based solely on the uncorrected recorded data can be inaccurate or erroneous, potentially leading to major misunderstandings about organismal physiology or behavior [13]. Here, we present two methods to improve the quality of data recovery in flow-through gas exchange systems.

### Background

Several methods have been employed to recover metabolic signals, including moving average [14], trend identification [15], Kalman-Bucky estimation [16], and stochastic deconvolution [17]. Among these methods, trend identification has shown a better performance in detecting long, near-constant CO_2_ emissions (rectangular pulses with a period of 32 min or longer), and the stochastic regularization method performs better in detecting smaller pulses of CO_2_ (rectangular pulses with periods of 16 min) [17]. However, the most well-used method for recovering the instantaneous gas exchange signal in small animals is the Bartholomew method, also known as the Z-transform (ZT) method [18]. Bartholomew et al. [18] first used this technique to determine the metabolic demand of preflight warm-up in sphingid and saturniid moths, an event with a duration on the order of 5 minutes. Since their study, this simple but powerful method has been widely used in a variety of other applications to measure the metabolic cost of transient phenomena in flow-through respirometry systems [1, 8, 9, 19, 20]. Independently, Woakes established a seemingly different method to recover the instantaneous gas exchange to study the correlation of the gas exchange and heart rate in swimming and diving ducks [21, 22]. However, Woakes’ method is mathematically identical to the ZT method, as we show in the Supporting Information (Appendix F in S1 File).

Despite the wide usage of the ZT method, there have been few attempts to probe the limits of the method or to enhance its accuracy [23–25]. The ZT method requires the application of a relatively simple mathematical process: the instantaneous metabolic signal is determined as a linear combination of the recorded data and its derivative. This method has two recognized problems. The first is that it uses the derivative of the data, and therefore any noise in the system will be magnified [1, 26]. A few analytical techniques have been developed to address this problem. Brychta et. al. [24] introduced a wavelet-based approach to remove the noise and retain the actual signals. Sun et. al. [23] showed that using a moving average filter can reduce the noise significantly. Chartrand [27] offered a general method to determine the derivatives of noisy data. These methods make it possible to sufficiently remove the noise, and have largely alleviated the noise problem.

However, the second problem, which is derived from the assumptions of the ZT method, remains largely unaddressed. The ZT method is constructed on the assumptions of immediate and uniform gas mixing in the chamber, and also the negligible effect of the finite length and diameter (ie. volume) of the pipes and gas analyzer on the metabolic signals. Using one or multiple fans inside the respirometry chamber [1, 23] can facilitate gas mixing, but the perfect mixing and negligible pipe length assumptions are not ideal [1, 13, 18, 26], and they need to be evaluated and replaced with more precise assumptions.

In an effort improve the quality of signal recovery, we tested the assumption of perfect mixing of the ZT method and then used our results to improve the method. First, we re-derived the ZT method’s equations and used this to establish a mathematical framework for finding its sources of imprecision. These mathematical tools provide the criteria needed to test the ZT method’s assumptions experimentally using a variety of volumes and flow rates of respirometry systems. We then use the developed mathematical framework to develop two new theoretical methods that enable the determination of instantaneous gas exchange signals with a much higher accuracy than previously possible. Lastly, we use experimental data to compare these methods with the ZT method, and demonstrate sub-second accuracy in a highly dynamical gas exchange using the analytical methods developed here.

## Methods

### ZT method

Here, we re-derive the equation first presented by Bartholomew et al. [18] from first principles, transforming it into a format that can be used to evaluate the assumptions of the method.

An open flow-through respirometry system drives air through the respirometry chamber containing the animal with air either pushed or pulled by a pump. The flow rate *F* is usually controlled with a flow meter. The fractional concentration of the gas of interest (O_2_, CO_2_, or H_2_O) is constant in the inlet flow, and the change (*c*) due to the animal is measured in the outlet flow (Fig 1). Here, we assume the inlet and outlet flows are equal and constant and that the respiration of the animal does not have a significant effect on the flow rate. Sometimes, when the gas of interest is CO_2_ or H_2_O, the inlet air is scrubbed to remove the gas of interest from the inlet air [5], but methodologically, this is not required. The animal inside the chamber consumes (O_2_) or produces (H_2_O/CO_2_) the gas of interest with the flow rate ${\dot{V}}_{X}$, where *X* refers to O_2_, CO_2_, or H_2_O. In the ZT method, we assume that gas mixing inside the chamber is immediate and complete, meaning that the concentrations of gases are instantly uniform across the chamber. In later equations, the gas exchange rate is divided by the inlet flow rate to provide a dimensionless rate, $u={\dot{V}}_{X}/F$.

To correct time-series respirometry data, Bartholomew et al. [18] provided the following equation (ZT method, see Appendix A in S1 File for more details):

$$u(k)=\frac{1}{Z}c(k+1)-\frac{1-Z}{Z}c(k)$$ (1)

where

$$Z=1-e^{-\frac{F}{V}T}$$ (2)

Here, *V* is the chamber volume, *T* is the sampling period, *k* is the sample number, and for small sampling times $Z=\frac{F}{V}T$.

Considering the conservation of mass (using the continuity equation), the concentration of the gas inside the respirometry chamber at time *t*, *c*(*t*), will change as follows:

c(t+dt)V=c(t)V+Fu(t)dt−Fc(t)dt (3)

where *c*(*t+dt*)*V* and *c*(*t*)*V* are the masses of the interest gas, and *t* is time. *Fu*(*t*)*dt* is the mass of the gas of interest that the animal infuses into the chamber, and *Fc*(*t*)*dt* is the mass that exits through the outlet flow during the interval *dt*. Eq 1 can be written as a first-order differential equation:

$$u(t)=c(t)+\frac{1}{F/V}\dot{c}(t)$$ (4)

and solved as follows:

$$c(t)=c(0)e^{-\frac{F}{V}t}+\frac{F}{V}\int_{0}^{t}u(\tau )e^{-\frac{F}{V}\tau }d\tau$$ (5)

where *c*(0) is the concentration of the gas at *t* = 0.

Eq 4 directly determines the instantaneous gas exchange rate (*u*) from the recorded data:

$$u(t)=c(t)+\frac{c(t+dt)-c(t)}{Z}$$ (6)

where $=\frac{F}{V}dt$, and it requires high sampling rate of the outlet gas. Discretizing Eq 4 for arbitrary sampling rates results in Eqs 1 and 2, which is known as the ZT method; this was derived in a different way in the original work [18] (see Appendices A and B in S1 File for details).

### The impulse response of a chamber

In linear system theory, one important way to analyze a linear system is finding and analyzing its impulse response, which is the reaction of the system to a very short unit impulse in a linear time-invariant (LTI) system. The key feature of the impulse response is that it contains all the dynamical characteristics of the system [28]. The output *c*(*t*) of any linear time-invariant (LTI) system from an arbitrary input *u*(*t*) can be determined by convolving the input with the impulse response of the system [29] if the system initially was at zero:

$$c(t)=\int_{0}^{t}u(\tau )h(t-\tau )d\tau$$ (7)

where *h*(*t*) is the impulse response of the system. By setting *c*(0) = 0 in Eq 5 and transforming it to the format of Eq 7, the impulse response of the ZT model can be determined to be $h(t)=\frac{F}{V}e^{-\frac{F}{V}t}$. Later, we compare this impulse response with the real impulse response of the system, which must be determined experimentally.

### Experimental setup

Two different experimental methods were used to find the impulse response of the respirometry chambers and to evaluate the new methods.

#### Setup 1

**Fig 2:**
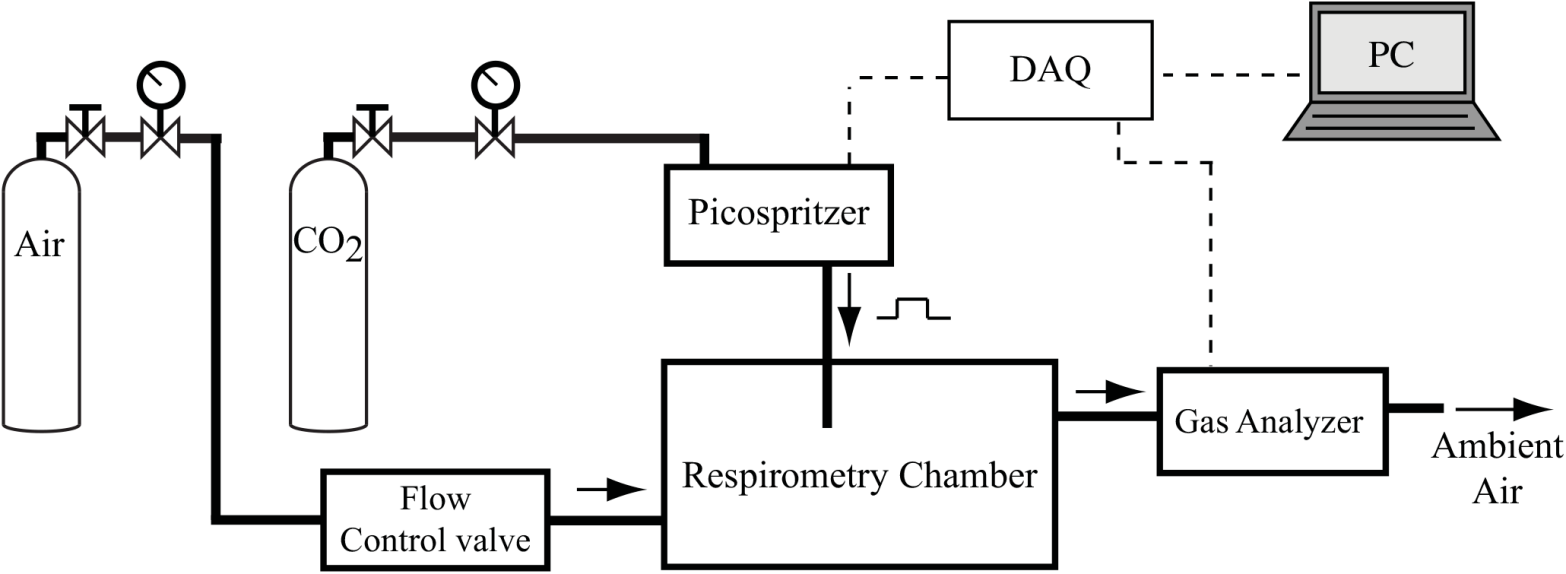
Experimental setup to determine the impulse response of a flow through respirometry system. The impulse response of each respirometry setup was found by infusing a pulse of CO_2_ with the duration of 100 ms by means of the picospritzer, roughly in the location where an animal would be. The recorded output was normalized to find the impulse response.

To determine the impulse response of a respirometry system for any given flow rate, a short pulse of CO_2_ was injected roughly in the location where the animal would be during experimental trials. The concentration of the output gas was then used to determine the impulse response of the system. Specifically, a short (100 ms) pulse of CO_2_ was injected with microinjector (Picospritzer III, Parker Hannifin, Precision Fluidics Division, NH, US) into the middle of the chamber, roughly reflecting the location from where the animal would release CO_2_, and the data were recorded with a gas analyzer (LI 7000 Li-Cor, Nebraska, USA, Fig 2). Then, we normalized the data by dividing it by the area below the data curve to find the impulse response of the system (see Appendix C in S1 File for details).

This protocol was repeated for three different respirometry chambers (volume 28, 125, and 600 mL) and five different flow rates (125, 250, 500, 1250, and 2500 mL/min). The 28 mL chamber was a custom 25×25×45 mm^3^ rectangular volume, and the 125 and 600 mL chambers were modified Fisher wash bottles (Fisher Scientific, MA, USA). The flow rate of pressurized CO_2_-free inlet air was controlled with a flow control valves (MFC 5850E, Coastal Instruments, Inc., NC). For each combination of chamber and flow rate, this process was repeated ten times, and the impulse response of the system was taken as the average.

To evaluate the technique of using fans to induce mixing [1, 23], we placed two relatively large fans (15x15mm 5V DC brushless, SEPA Europe GmbH, Freiburg, Germany) inside the 28 mL chamber, close to the inlet and outlet ports, and determined the impulse response at flow rates of 125, 250, 500, 2500 mL/min.

#### Setup 2

**Fig 3:**
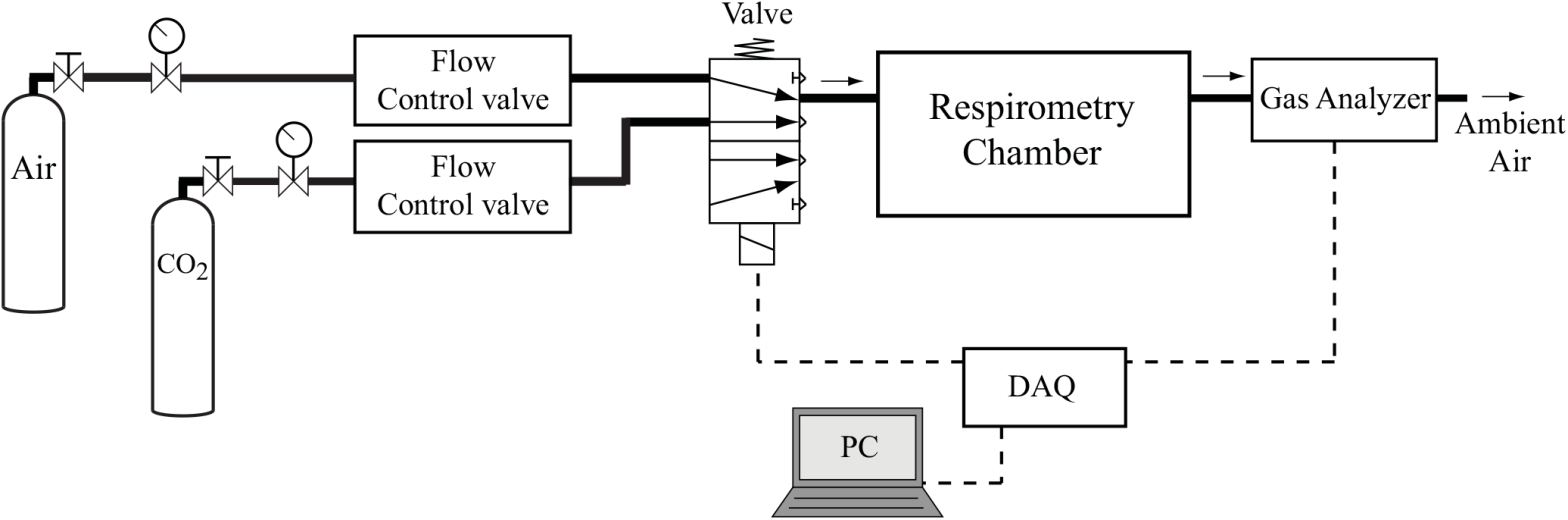
Experimental setup to evaluate the methods. A high-speed valve switches the inlet flow between pure air and 100 ppm CO_2_. The concentration of CO_2_ in the outlet gas is measured in the gas analyzer.

Infusing CO_2_ into to the inlet flow for long periods of time will change the flow rate of the system, invalidating the LTI system assumption (i.e., Eq 3 will not be valid). Therefore, to evaluate the methods, we used a high-speed pneumatic valve (MHE2-MS1H-5/2-M7-K, Festo, NY, USA) to switch between the CO_2_-free air and 100 ppm CO_2_ gas (Fig 3) at the inlet. In this way, by maintaining the same flow rate for both gases, the inlet flow rate was kept constant. The valve was controlled with LabVIEW and a data acquisition device (NI 9472, National Instruments, Austin, USA). Because the new setup has a different impulse response than setup 1 (with microinjector), we determined its impulse response with the same process and used it in the data recovery algorithms.

### Experimental evaluation of the methods

After recording the input signal and the output CO_2_ concentration, we applied the ZT method and the new methods to the data to recover the input signal. The estimation of the input signal $\hat{u}(t)$ was compared with the true input signal *u*(*t*), and the error of the method was found as follows:

$$\text{ITAE}=\int_{0}^{\infty }\left|\overset{⌢}{u}(t)-u(t)\right|dt$$ (8)

where ITAE is Integral Time Absolute Error (ITAE = $\int_{0}^{\infty }|e|dt$).

Two sets of experiments were used to evaluate and compare the methods:

#### 1- CO_2_ infusion in different frequencies

In the first set of experiments, the 28 mL respirometry chamber was used and the high-speed pneumatic valve was used to switch between CO_2_-free air and 100 ppm CO_2_ gas before the respirometry chamber with controlled frequencies. To probe the accuracy of the methods, the frequency of switching between air and CO_2_ was set to 0.1, 0.15, 0.25, 0.5, and 1 Hz. Three pulses of CO_2_ at each frequency were injected into the chamber. This experiment was repeated for two flow rates of 250 and 500 mL/min and the data were recorded at 10 Hz.

#### 2- Impulse response curve

In the second set of experiments, we aimed to recover a very short pulse of CO_2_ from the respirometry data using each method. In the previous trials, the shortest possible input was used to determine the impulse response of the respirometry chambers at different flow rates. Therefore, we used the impulse response data from the 28 mL chamber with the flow rate of 500 mL/min.

### Experimentally tuning the constants of the methods

The ZT method uses a constant parameter (*Z* in Eqs 1 and 2) to determine the true gas exchange signal. The EZT and GZT methods also contain some constant parameters that can be determined analytically or experimentally. Previous studies have shown that a theoretically-determined *Z* does not work accurately in experiments and should be tuned experimentally [1, 18, 26]. We found the parameters for the new method with experiments as well. For this purpose, the varying input *u*(*t*) and output *c*(*t*) were recorded experimentally and then used to determine the parameters. In the first experiment, we used the 28 mL chamber and the flow rates of the air and CO_2_ line were set to 250 mL/min (Fig 3). Using the pneumatic valve, three pulses of CO_2_ with durations of 200 ms and offset two seconds apart were injected into the chamber. The data were recorded at a sampling rate of 10 Hz. We then repeated the same experiment using a flow rate of 500 mL/min. We used these data to find the constant parameters of the each method in this paper (ZT, EZT, and GZT methods).

The coefficient of each method can be found by minimizing the error (ITAE) between the estimated input and the real known experimental input, *u*(*t*), using the optimization algorithm ‘fminsearch’ function in Matlab (R2013a, MathWorks, USA) (Eq 8).

### Noise reduction

Respirometry systems operate like a low-pass filter, smoothing out fast changes in the gas exchange signals. Therefore, the inverse process, recovering the true gas exchange signals from the recorded data, can be considered as a high pass filter, which amplifies noise. In this study, we used a moving average with span of 10 data points to reduce the noise in the recorded data before applying the recovery methods. In the two methods that we will discuss in detail (ZT and EZT methods), one or more derivatives of the data must be taken. To reduce the noise amplification that occurs when taking derivatives, we filtered the noise with a moving average after taking each derivative.

All input recovery methods magnify noise. We applied each method to the recorded signal at time points when there was not any CO_2_ in the chamber, and all the recorded data is noise. We determined the root mean square of the recovered signal, which is pure noise, and considered twice this value as a threshold. In the evaluation experiments, we considered any signal below this threshold as noise and applied a stronger filter for these signals (moving average with the span of 50 points).

### Case study: objectively determining spiracular opening in beetles

An important question in studying gas exchange in insects is determining the status of the spiracles and the durations of closed and open phases [13]. In many insects, it is difficult to unequivocally determine the status of the spiracles, and in some cases an indirect method is employed to objectively determine the duration of the closed and open phases of the spiracles [30]. Here, we measure ${\dot{V}}_{CO_{2}}$ of the animal and then compare it with a specified threshold using the same method as described by Contreras et al. [30]. This cutoff threshold is defined by the ${\dot{V}}_{CO_{2}}$ under the condition of hyperoxia. It is known that under hyperoxia, the duration of the closed phase of spiracles significantly increases. Therefore, to determine the cutoff threshold, ${\dot{V}}_{CO_{2}}$ of the animal under hyperoxic condition (100% oxygen) was measured. Then the average of the lowest continuous ${\dot{V}}_{CO_{2}}$ values during a two-minute trial is multiplied by a factor of four and considered the cutoff threshold. If ${\dot{V}}_{CO_{2}}$ is above this threshold, then the spiracles are assumed to be open, otherwise they are considered closed [30].

In this experiment, we used *Zophobas morio* beetles (Carolina Biology Supply, NC, USA) to determine the duration of opening phase. Animals were maintained in a terrarium containing a mixture of sand and soil and were provided bran meal and water *ad libitum*. The CO_2_ production rate of a *Z*. *morio* adult was measured in the 28 mL respirometry chamber with an inlet flow rate of 500 mL/min for an hour at 22°C. Then, the inlet air was replaced with 100% oxygen for 20 minutes to maintain the hyperoxic condition to find the cutoff threshold.

## Results

### Analyzing the Z-transform method

Comparing Eqs 5 and 7 shows that the impulse response of the respirometry system in the ZT model is a first-order exponential decay, expressed as:

$$h(t)=\frac{F}{V}e^{-\frac{F}{V}t}$$ (9)

**Fig 4:**
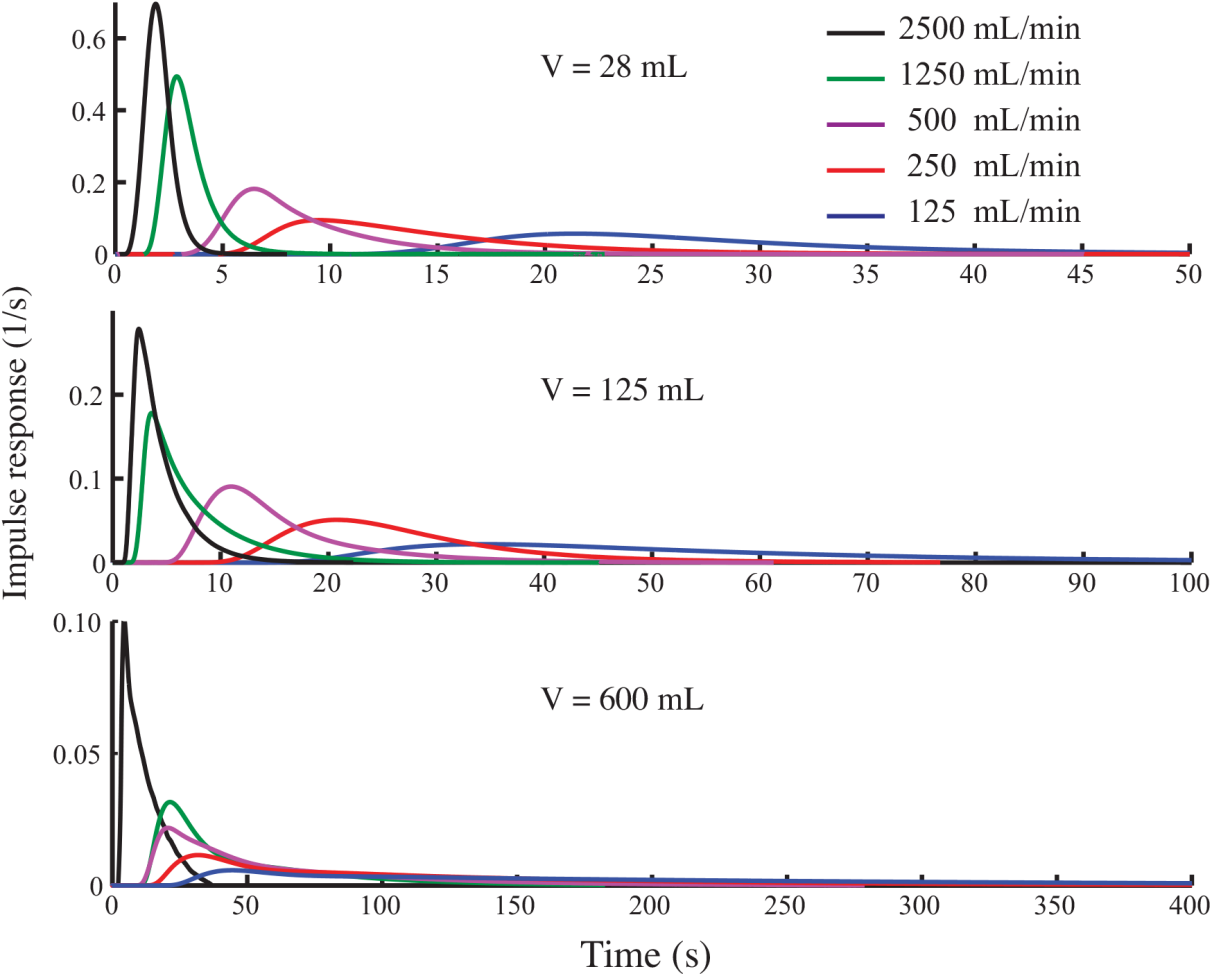
Impulse responses of three respirometry chambers with five different flow rates. None of the impulse responses are in the form of pure exponential decay.

**Fig 5:**
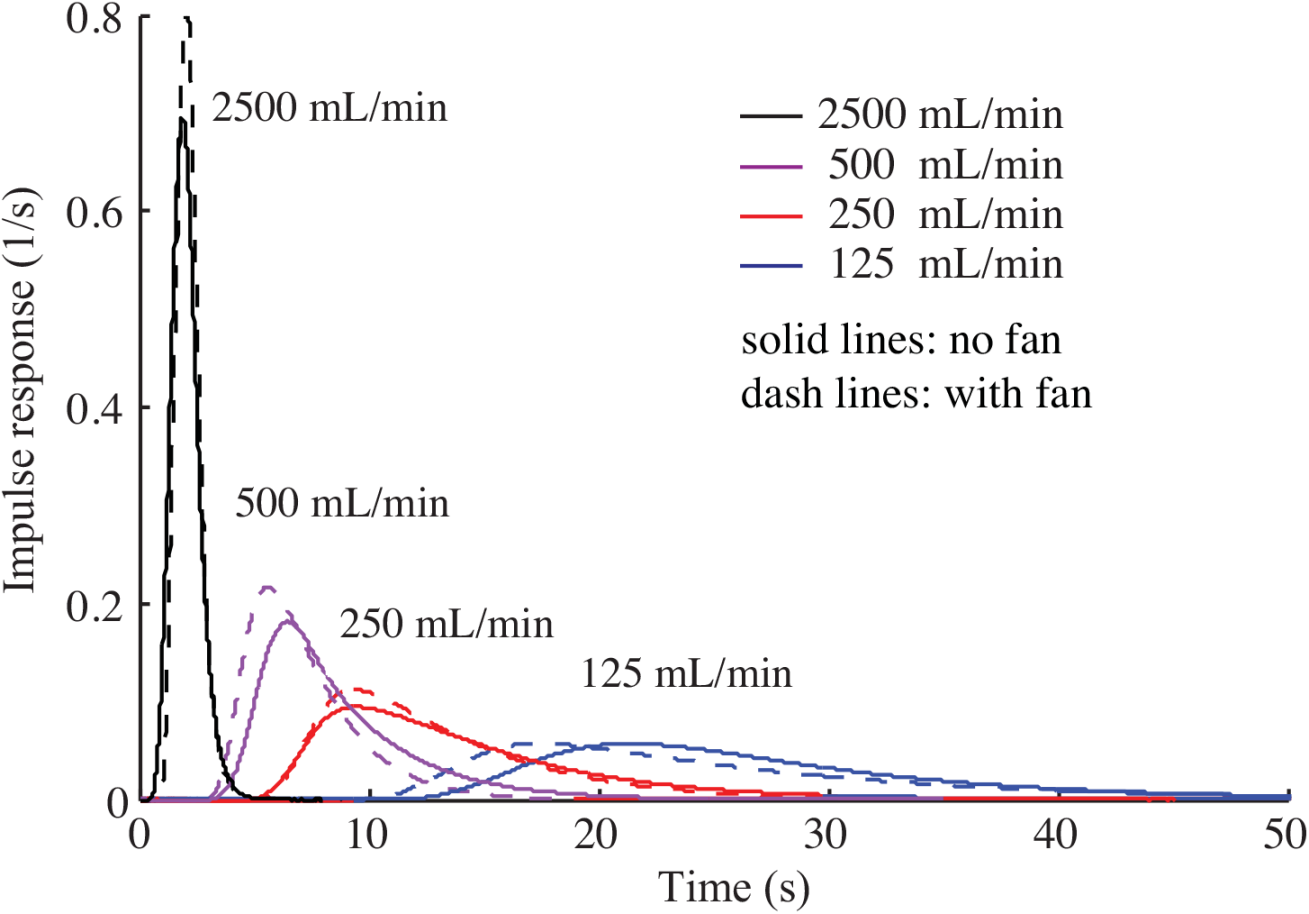
The effect of fans on the impulse response of the system. Even after using a fan the impulse responses do not approach exponential decay. In this experiment the impulse responses for a 28 mL respirometry chamber in different flow rates is determined when the embedded fans within the chamber are on and off.

However, our experimentally-determined impulse responses of the respirometry systems are not consistent with exponential decay (Fig 4). Using fans within the respirometry chamber improves the mixing, but even in the presence of fans, the impulse responses are still not close to a first-order exponential decay (Fig 5).

### A new model for respirometry impulse response

After determining the impulse response of different combinations of chambers and flow rates, we explored several different functions in an attempt to better model the response. We found that the shape of the impulse response can be described more accurately using the expression *α t* ^*m*^ *e* ^−*βt*^ rather than that of a simple exponential decay, expressed as *α e* ^−*βt*^. Here *m*, *α*, and *β* are parameters of the system; *α* is a coefficient used to normalize the impulse response and is not independent from the other two parameters: $\alpha =\frac{\beta^{m+1}}{m!}$ (see Appendix C in S1 File for details).

**Fig 6:**
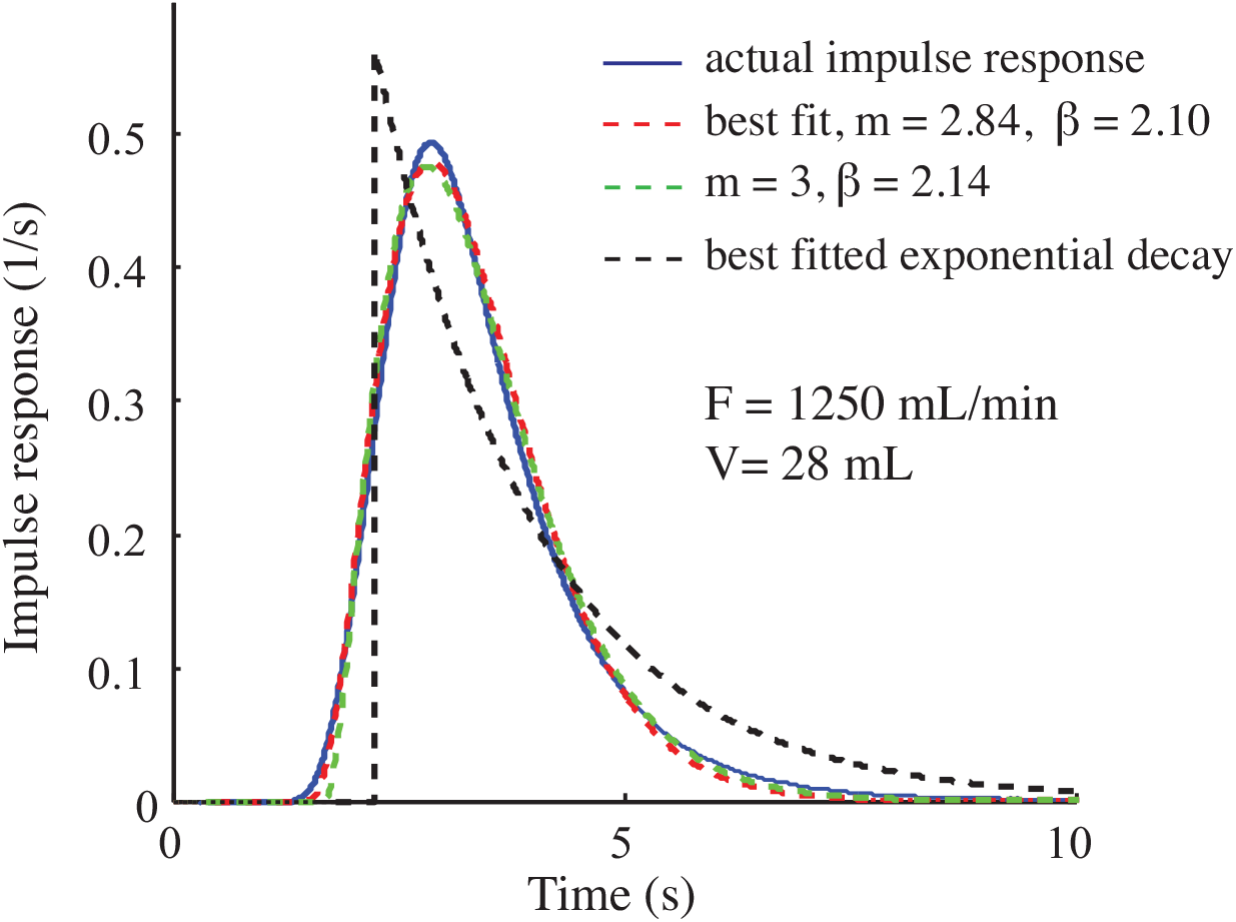
Fitting different curves to the impulse response. The curves with new equations fit more accurately to experimentally determined impulse response than the exponential decaying curve (black).

The impulse response also contains a pure delay, which can be eliminated by simply shifting the data (Fig 6). Table 1 shows the value of the parameters *m*, *β*, and the delay for each impulse response. Later, we demonstrate the governing dynamical equations of the system can be derived if *m* is an integer number. In general, *m* can be any positive real number, but forcing it to be an integer does not significantly increase the error. In the next section, we show that the first *m+*1 derivatives from the recorded data are needed to recover the actual metabolic patterns. Therefore, we try to minimize *m* without losing accuracy. As an example, the actual impulse response of the 28 mL chamber with flow rate of 1250 mL/min, which is determined experimentally, is compared with three other estimated impulse responses (Fig 6): 1) the best fit exponential decay (the ZT method), 2) the best fit new model (*α t* ^*m*^ *e* ^*−βt*^) with a real number for *m*, 3) the best fit new model (*α t* ^*m*^ *e* ^*−βt*^) with *m* restricted to be an integer. The parameters of the models were determined by minimizing the ITAE. The ITAE of the models is 39.3%, 6.9%, and 7.1% respectively. These results indicate that the new model fits the real impulse response more accurately than the simple impulse response. It also shows that the error due to forcing the parameter *m* to be an integer number is negligible (see Fig 5 and Table 1).

**Table 1:** Parameters of the impulse responses. Experimentally-determined impulse responses of three respirometry chambers in five different flow rates were modeled in four ways. In the first three models, αt^m^e^−βt^ has been fitted to the data. Here m, α, and β are constant parameters that are needed to fit this curve (αt^m^e^−βt^) to the experimental data (see text for details). In the first one, *m* is considered as a real number. In the second, one we restricted the *m* to be an integer and then found the parameters. In the third one, we decreased *m* to the lowest integer number without having more than 10% ITAE ($\int_{0}^{\infty }|e|dt$). In the last one, we forced *m* to be zero in order to recover the ZT model.

		Best fit curve			EZT method						ZT method	
V	F				Best integer *m*			Small *m*			(*m = 0*)	
(mL)	(mL/min)	*m*	*β* (1/s)	*δ* (s)	*m*	*β*	*δ*	*m*	*β*	*δ*	*β*	*δ*
	125	1.65	0.188	12.75	2	0.206	12.05	1	0.15	14.16	0.066	16.62
	250	0.97	0.246	5.87	1	0.249	5.82	1	0.249	5.82	0.1094	7.204
28	500	1.16	0.49	4.08	1	0.459	4.19	1	0.459	4.19	0.2018	5.025
	1250	2.85	2.089	1.5	3	2.142	1.47	2	1.761	1.68	0.5657	2.236
	2500	14.2	6.58	0.29	14	6.53	0.29	3	3.045	0.79	0.8091	1.338
	125	0.86	0.054	18.9	1	0.057	18.09	1	0.057	18.09	0.2441	23.95
	250	1.83	0.178	10.58	2	0.185	10.2	1	0.134	12.51	0.0575	15.15
125	500	1.12	0.246	6.27	1	0.234	6.44	1	0.234	6.44	0.1011	8.0
	1250	0.35	0.291	2.46	0	0.189	2.74	0	0.189	2.74	0.1892	2.74
	2500	0.67	0.592	1.42	1	0.699	1.26	1	0.699	1.26	0.3057	1.77
	125	0.026	0.005	29.36	0	0.0035	29.75	0	0.0035	29.75	0.0035	29.75
	250	0.048	0.011	19.91	0	0.0102	20.46	0	0.0102	20.46	0.0102	20.46
600	500	0.144	0.027	13.83	0	0.0217	14.65	0	0.0217	14.65	0.0217	14.65
	1250	0.206	0.042	14.36	0	0.0313	15.33	0	0.0313	15.33	0.0313	15.33
	2500	0.204	0.131	3.03	0	0.0969	3.26	0	0.0969	3.26	0.0968	3.26

### Extension of ZT (EZT) method

The governing equation of a LTI system with an impulse response of *α t* ^*m*^ *e* ^*−βt*^ is as follows:

$$u(t)=\frac{1}{\alpha m!}e^{-\beta t}\frac{d^{m+1}}{dt^{m+1}}\left(e^{\beta t}c(t)\right)$$ (10)

See Appendix D in S1 File for details. This equation can be expanded to:

$$u(t)=\sum_{k=0}^{m+1}a_{k}\overset{\left(k\right)}{c}(t)$$ (11)

where $\overset{\left(k\right)}{c}(t)$ is the *k* ^*th*^ derivative of *c*(*t*) and

$$a_{k}=\frac{1}{\beta^{k}}\left(\begin{array}{c} m+1\\ k \end{array}\right)\;(k=0,\;1,\ldots ,\;m+1)$$ (12)

Eq 10 determines the instantaneous gas exchange from the recorded data and its derivatives. From this equation, it can be seen that *m*+1 derivatives of the data are required to recover the actual signal. The ZT method (Eq 4) is included as a special case, by setting *m* = 0 and $\alpha =\beta =\frac{F}{V}$. Here, only the first derivative of the data is used to recover the instantaneous gas exchange signal.

This method uses *m* derivatives of the recorded data, and therefore potentially magnifies the noise. Here we use three techniques to reduce the noise amplification. First, we use noise-robust techniques to take derivatives from the noisy data (see Appendix E in S1 File). It is also possible to take derivatives from the data and then smooth them using a moving average, which we found to be equally effective. Second, we filtered the noise after each derivation. Lastly, we used approximate fits to the impulse responses with smaller *m* (‘Small *m*' column in Table 1). As Table 2 shows, most of the impulse responses can be approximately modeled using *m* ≤ 3.

**Table 2:** Estimated parameters of ZT and EZT methods using Eqs 2 and 11 and from experimental data.

	F	Parameter		
Method	(mL/min)	estimation method	a_1_	a_2_
	250	Eq 2	6.72	-
ZT	250	Experiment	12.2	-
	500	Eq 2	3.36	-
	500	Experiment	5.3	-
	250	Eq 12	8.03	16.13
EZT	250	Experiment	12.3	41.2
	500	Eq 12	4.36	4.75
	500	Experiment	5.2	5.1

### Generalized ZT (GZT) method

The Bartholomew method (Eq 4) and its extension (Eq 11) show that the instantaneous gas exchange can be written as a linear combination of the data points and their derivatives. In discrete form, the instantaneous signal at any point is a linear combination of several data points around it. Here, we extend this idea and present a simple but efficient numerical method to recover the true metabolic signals. Because the input at any time *k* (in discrete form) affects the future outputs for some duration, the conjecture is that the input (instantaneous gas exchange signal) can be estimated by a linear combination of *N* future outputs:

$$u(k)=\sum_{j=0}^{N}a_{j}c(k+j)$$ (13)

where *N* is equal to or less than the length of impulse response. All parameters (*a*_*j*_) depend on the dynamical characteristics of the respirometry system and must be determined experimentally. We call these parameters ‘calibration coefficients’. To find the calibration coefficients, a series of known inputs should be injected into the chamber and the output recorded. Then, the input and output data points should be placed in Eq 13 and solved to determine the unknown calibration coefficients *a*_0_ to *a*_N_, using the least squares method to minimize the error:

$$a={\left(C^{T}C\right)}^{-1}C^{T}u$$ (14)

where *a* = (*a*_0_,⋯,*a*_*N*_)^*T*^, *u* = (*u*(0),⋯,*u*(*n*))^*T*^, and *C* is a matrix composed of the output data points:

$$C=\left(\begin{array}{c} C_{0}\\ C_{1}\\ \vdots \\ C_{n} \end{array}\right),\;C_{j}={\left(c(j),\cdots ,c(j+N)\right)}^{T}$$ (15)

The GZT method can be implemented in practice using the following steps:

*step-1*: Infuse CO_2_ gas with a known arbitrary pattern (*u*(*t*)) into the respirometry chamber and record the output signal (*c*(*t*)). The length of the input, *n*, should be several times larger than the length of the impulse response to produce the most accurate results.

*step-2*: Construct the matrix *C* using Eq 15 and then use it in Eq 14 to calculate the calibration coefficients (*a*).

*step-3*: To determine the instantaneous gas exchange signal (u) in a real experiment, use the recorded data from the gas analyzer and the calibration coefficients (vector *a*) in Eq 13.

To help a researcher implement these steps in practice, a MATLAB code is provided (S2 File) and explained in the Supporting Information (Appendix G in S1 File). This code is also permanently available online at GitHub [31]; improvements to the code and a planned port to Python will be implemented and posted there in the future.

### Experimental verification

#### Determining the parameters

Table 2 provides estimated parameters using Eqs 4 and 12 and compares them with the experimentally determined parameters for the ZT and EZT methods. The coefficient in the generalized method was determined by using the Eq 14. N in this equation was selected by trial and error (N = 230), by applying the method with different N’s on calibration trials data and minimizing the input estimation error.

#### Recovering frequently changing respirometry signals

**Fig 7:**
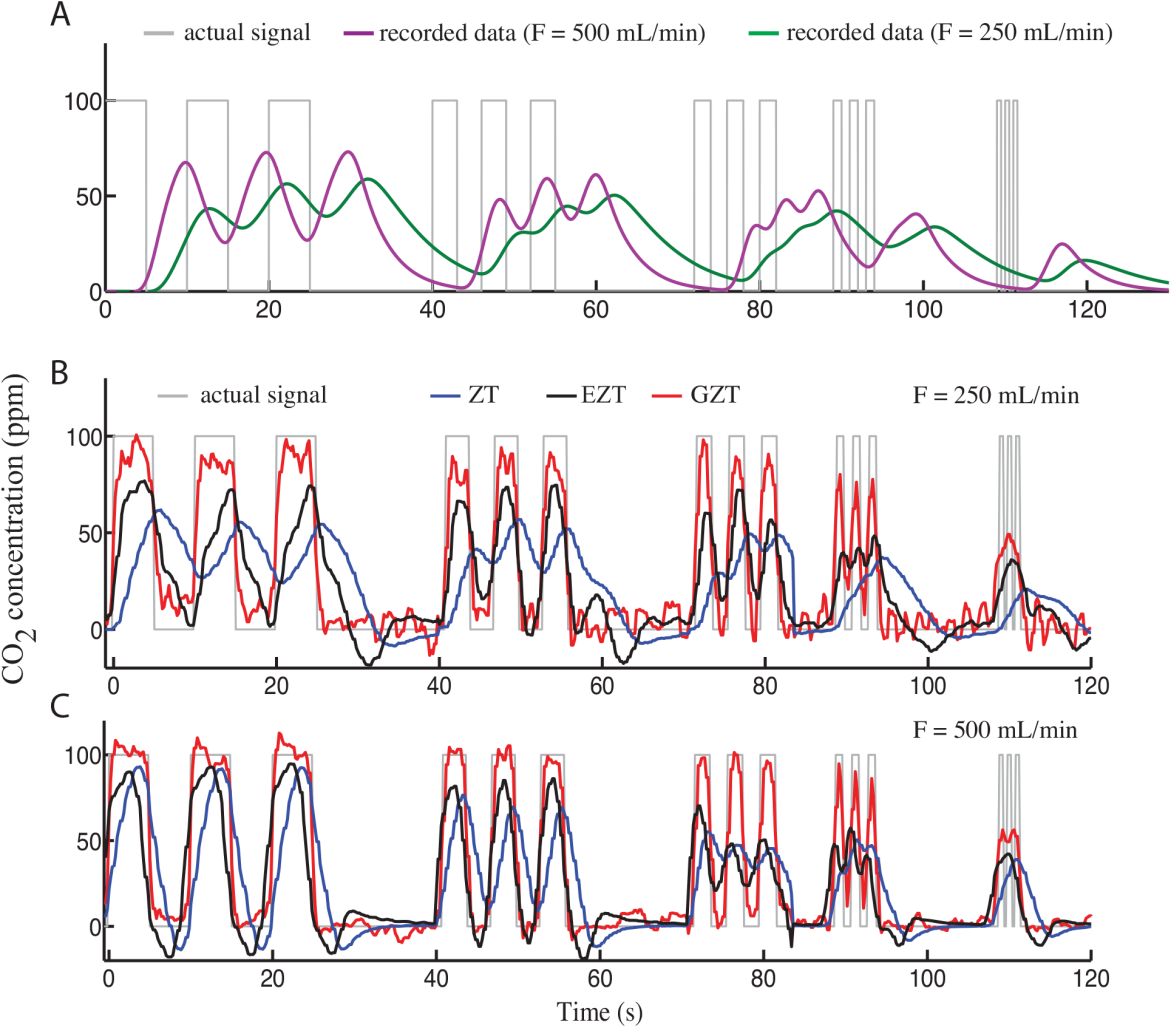
Comparing different methods for recovering the CO_2_ input. CO_2_ was infused with rectangular pulses with different frequency and duration into the respirometry chamber in two flow rates of 250 and 500 mL/min and the output was recorded (A). ZT, EZT, and GZT methods were used to recover the CO_2_ inputs from the recorded data (B and C). The results indicate that the precision of GZT method is significantly higher than the ZT and EZT methods and the EZT is more precise than the ZT method.

In the first evaluation experiments, CO_2_ was infused into the chamber with several different frequencies. The input signal was estimated by using all three described methods. The real input and recovered data using the ZT method, the EZT method, and the GZT method are compared in Fig 7. In general, the performance of all the methods decreases with increasing the frequency (Table 3). The IATE of the EZT method was less than the ZT method in all the tests, and the performance of the GZT method was better than both ZT and EZT methods (Fig 7, and Table 3). The GZT method especially improves the results for high-frequency gas exchange, which is particularly difficult to recover. The recovered signal by this method was synchronized with the real input even for the 0.5 Hz pulses (Fig 7); however, the recovered 1 Hz signal is over-damped. The EZT recovered signals are synchronized with the true input signals at 0.1 and 0.167 Hz and show similar peaks at 0.25 Hz, but for higher-frequency inputs, the recovered signal is damped. ZT recovered signals are synchronized only in the lowest frequency input (0.1 Hz) and 0.167 Hz with high flow rate (500 mL/min). The estimations of this method are over-damped in higher frequencies. Collectively, in terms of having minimum error of signal recovery, the GZT method performs better than ZT and EZT methods and the performance of the EZT method is better than the ZT method (Table 3).

**Table 3:** Normalized ITAE to the area of the input signal of the recovered signals in different frequencies.

	F	Normalized ITAE				
Method	(mL/min)	0.1 Hz	0.167Hz	0.25 Hz	0.5 Hz	1 Hz
ZT		1.1767	1.1667	1.1683	1.5375	1.6032
EZT	250	0.7577	0.6889	0.8073	1.2268	1.3170
GZT		0.3269	0.4096	0.4757	0.7862	1.2056
ZT		0.4500	0.7904	0.9496	1.1264	1.4902
EZT	500	0.3724	0.5400	0.9004	1.2114	1.4116
GZT		0.2699	0.4855	0.5010	0.7204	1.3549

**Fig 8:**
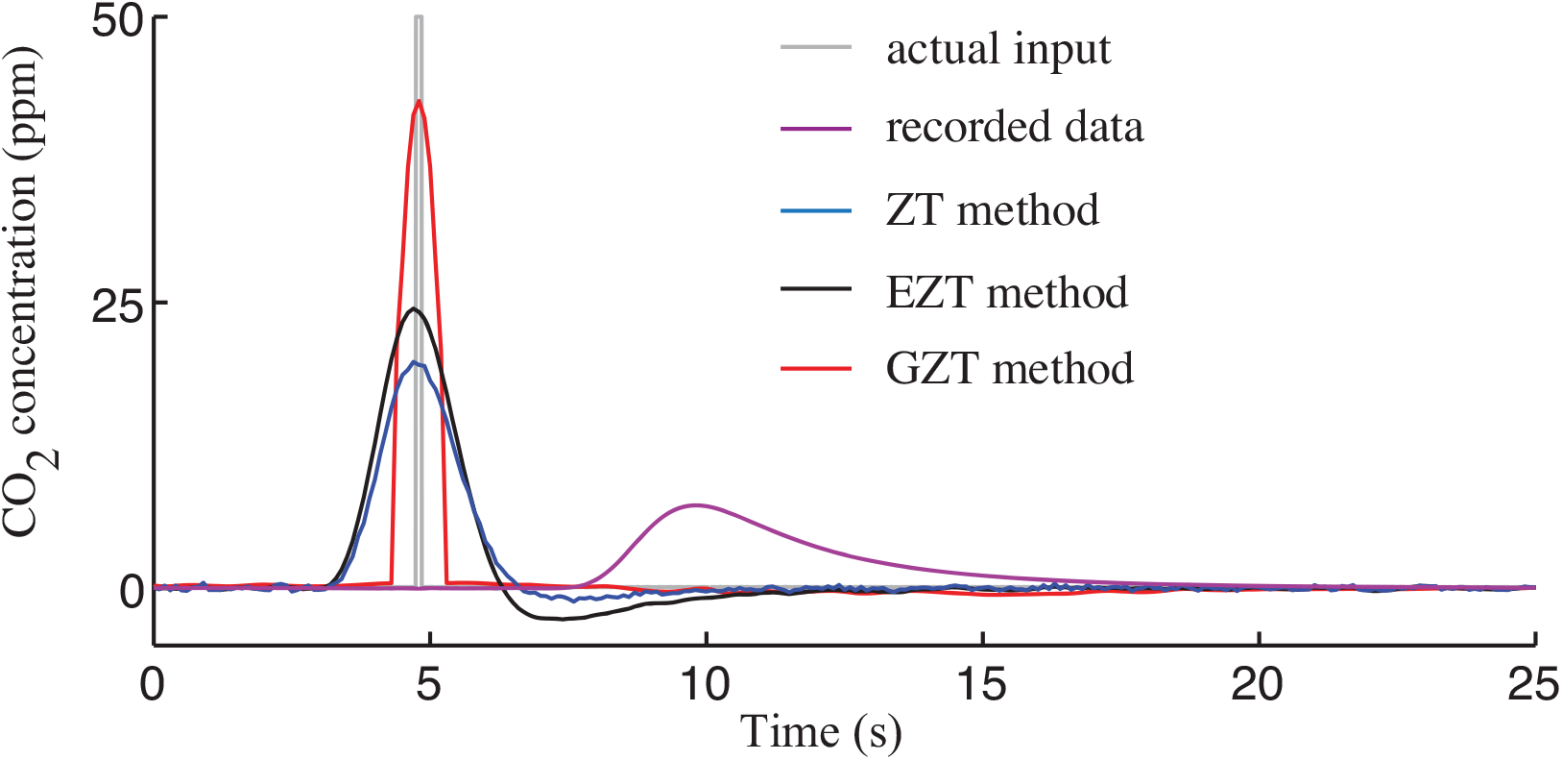
Comparison of ZT, EZT, and GZT methods. The injected input of 100 ppm CO_2_ with duration of 200 ms into the 28 mL respirometry chamber is estimated from the output signal using different methods.

In the next evaluation, the methods were used to recover a very short pulse of CO_2_. All of the methods were able to recover short pulses, but with different precisions (Fig 8). The normalized IATE for the ZT, EZT, and GZT is 2.3702, 2.2332, and 1.7781, respectively, which indicates that the GZT method is more accurate than the other two.

#### Case study: objectively determining spiracular opening in beetles

**Fig 9:**
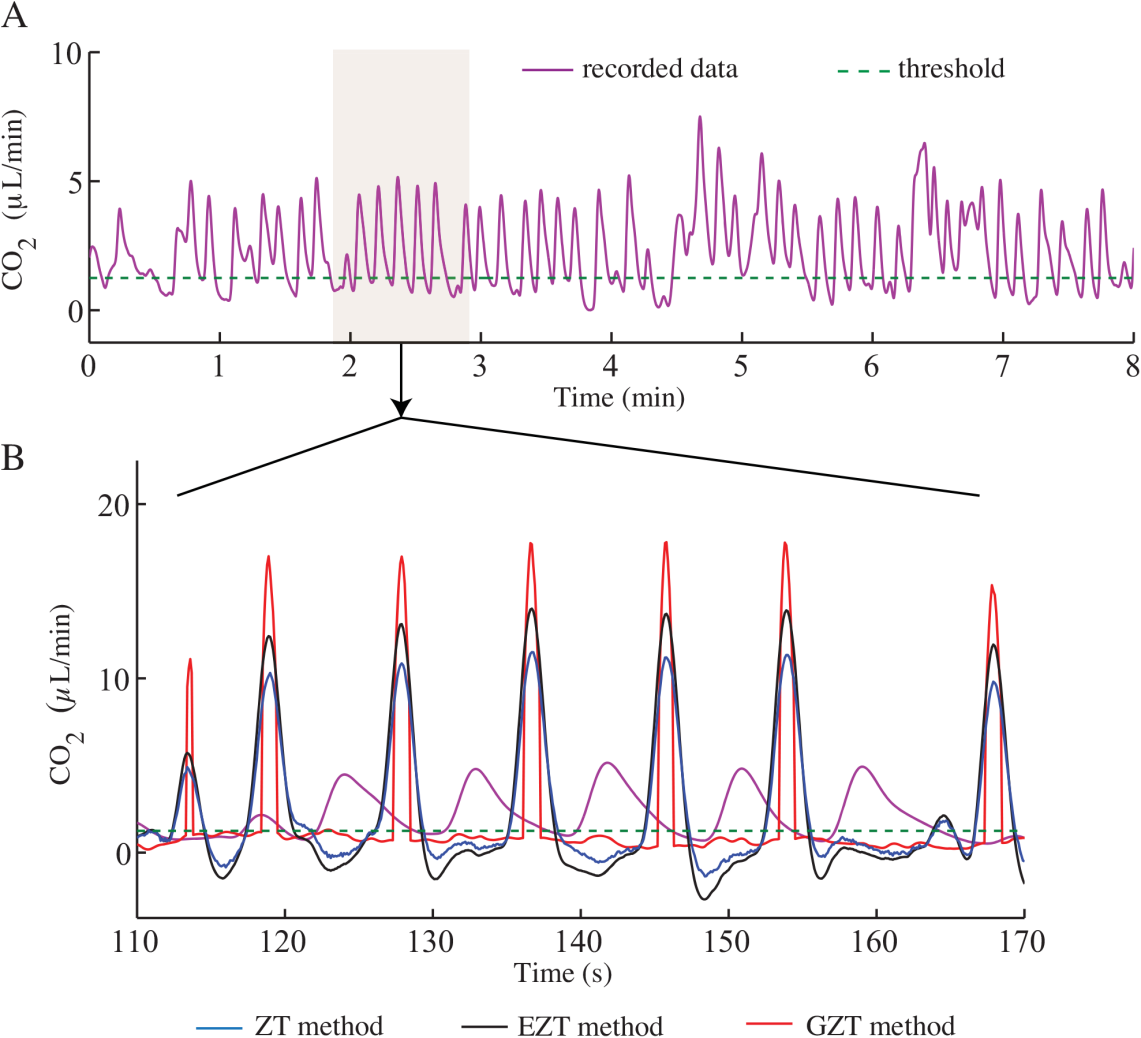
Recovery of the actual metabolic rate of a beetle. ZT, EZT, and GZT methods were applied on the recorded respirometry data of a *Zophobas morio* adult and the recovered instantaneous signals compared with a threshold line to find the open and closed phase of the spiracles.

To demonstrate the real-world effect of higher precision signal recovery, we recorded the CO_2_ release rate of *Zophobas morio* to identify the opening and closing phase of its spiracles. To objectively determine the opened or closed status of spiracles, we compared the CO_2_ production rate of the animal with the threshold, and whenever the signal was above the threshold, we considered the spiracles to be open. The threshold CO_2_ rate for the animal was found to be 1.12 μL/min from the hyperoxic experiment. The ZT, EZT, and GZT methods were applied to the data to calculate the duration of opening and closing phases (Fig 9). The percentage of time that the spiracles were open was determined by dividing the summation of the open times by the duration of the experiment (Table 4). The calculated open phase durations can vary greatly across methods (Fig 9, Table 4).

**Table 4:** Calculated opening phase based on different recovered respiratory data.

Method	Opening phase (%)
Raw data	70.2
ZT	44.6
EZT	41.1
GZT	34.2

## Discussion

In this work, we experimentally determined the impulse response of three different respirometry chambers and showed that the impulse response can be modeled in the general form of *α t* ^*m*^ *e* ^*−βt*^. The extension of ZT (EZT) method, which is based on a new model of the impulse response, demonstrated an improvement in estimation of the instantaneous gas exchange up to 41% (Table 3, Figs 7 and 8). The performance of the generalized ZT (GZT) method was considerably higher than the previous methods, with experiments demonstrating an enhancement up to 72% (Table 3, Figs 7 and 8).

The GZT method is not only more accurate than ZT and EZT, but it is independent of the shape of impulse response, which means that it should be broadly applicable to any respirometry system. The impulse response of a system contains all the dynamical characteristics of the system, and many parameters affect its shape. In experimental respirometry systems, the geometry of the chamber and tubing, flow rate, diffusion rate of the gas, and the dynamical properties of the gas analyzer are involved in shaping the impulse response. The new mathematical representation of the impulse response that we introduced in this paper, *α t* ^*m*^ *e* ^*−βt*^, more accurately fits the empirical data from our respirometry systems than does the previous version, *α e* ^*−βt*^. However, we cannot claim that this improvement represents the global form of the impulse response for any arbitrary respirometry system, because it was determined experimentally from a limited combination of our respirometry chambers and flow rates. Therefore, the method that was developed based on this equation, EZT, might not be suitable for all flow-through respirometry systems. Instead, we recommend the GZT method as a safer method to use.

Although the precision of the methods was demonstrated experimentally, there still remain multiple major issues to address in future studies: 1) A general algorithm to determine the uncertainty of the presented methods does not yet exist; therefore, the first open question would be how to establish such an algorithm. 2) An algorithm to choose the optimum sampling rate (1/*T*) is not developed. To recover high-frequency gas exchange patterns, the sampling rate should be relatively high. However, increasing the sampling rate lowers the signal-to-noise ratio, and thus can adversely affect the derivatives of the data, where errors are amplified by greater noise. Therefore, it would be useful to develop a method to determine optimum sampling rates for real systems. 3) In the GZT method, the choice of *N* in Eq 13 is done by trial and error, and different choices for *N* may lead to different results. A defined algorithm for finding *N* needs to be developed.

In addition to the ZT method, other methods such as a moving average [23], trend identification [15], Kalman-Bucky [16], and stochastic deconvolution with regularization parameter [17], have been used before to estimate the actual pattern of the gas exchange in room calorimeters. The stochastic deconvolution method has been shown to have a better performance in recovering the transient response of whole-body calorimeters in comparison with moving average, trend identification, and Kalman-Bucky [17]. To the best of our knowledge, these have not been compared with the ZT method. In a previous study [17], the stochastic regularization method was applied to a respirometry chamber with the volume of a 16.6 m^3^, flow rates of 50–150 L/min, sampling rate of 1/min, and the impulse response of the system was considered to be an exponential decay. This method recovered periodic rectangular CO_2_ pulses with periods down to 16 minutes. The stochastic deconvolution method uses high-dimensional square matrices with the size of the length of the recorded data. Therefore, applying this method to data from long experiments or with high sampling rates may be difficult or require modifications, resulting from the large size of the data set. But the ZT, EZT, and GZT methods can be applied to data sets of any size. In the examples we presented here, we recovered rectangular CO_2_ pulses with periods down to 4 seconds. But these results are not comparable; because the methods have been applied on two different systems, with different chambers, flow rates, and sampling rates. Lastly, we have not yet not compared our new methods (EZT and GZT) with the stochastic deconvolution method; this will be conducted in a future study.

The presented methods are suitable for any flow-through respirometry system of any size and may also be applicable in indirect calorimeters, in which other methods have been used to recover their original signals [23, 32]. Moreover, because the GZT method works for any linear system with any impulse response, it can potentially be used to provide a more precise input estimation for previously published data across physiological systems. In general, in many physiological processes such as hormonal secretion, production of glucose, and oxygen consumption it is difficult or impossible to directly measure the signal of interest, and typically they have been measured indirectly [1, 13, 16–18, 33–40]. In all of these examples, the relationship between the input signal (the signal of interest) and the output signal, which is possible to measure, can be estimated with a linear differential equation. Therefore the ZT, EZT, and GZT methods might be broadly useful for input estimation of these biological systems.

Previous respiratory work has applied signal recovery to analyze slow phenomena (on the order of minutes) [1, 17, 26]. However, there could be much more valuable information if the fast dynamical signals were also recovered from the recorded data. For instance, there are many fast processes that could affect the respiration of animals. Examples of events in insects that occur on the second or sub-second timescale include abdominal pumping [41], compression of tracheal tubes [10, 11], and opening of spiracles [42]. To study the effect of these dynamical behaviors in insects, it is necessary to recover the sub-second signals from the respirometry data, which we have shown is possible to do more accurately with our new methods. Overall, these developments demonstrate that experimentalists can extract more information from their data, leading to a truly time-resolved understanding of the respiratory patterns of animals.

## Supporting Information

S1 File Mathematical and experimental details of the method, including seven appendices (A-G). (PDF)

S2 File Matlab code and sample data files to implement the GZT method, to enable researchers to apply the method with their own experimental data. (ZIP)

## References

[1] Lighton J, Halsey L. Flow-through respirometry applied to chamber systems: pros and cons, hints and tips. Comp Biochem Physiol, A: Comp Physio. 2011;158(3):265–75. 10.1016/j.cbpa.2010.11.026 21134483

[2] Depocas F, Hart JS. Use of the Pauling oxygen analyzer for measurement of oxygen consumption of animals in open-circuit systems and in a short-lag, closed-circuit apparatus. J Appl Physiol. 1957;10(3):388–92. 13438789 10.1152/jappl.1957.10.3.388

[3] Fedak MA, Rome L, Seeherman HJ. One-step N2-dilution technique for calibrating open-circuit VO2 measuring systems. J Appl Physiol. 1981;51(3):772–6. 7327980 10.1152/jappl.1981.51.3.772

[4] Withers PC. Measurement of VO2, VCO2, and evaporative water loss with a flow-through mask. J Appl Physiol. 1977;42(1):120–3. 833070 10.1152/jappl.1977.42.1.120

[5] Lighton JR. Measuring Metabolic Rates: A Manual for Scientists: A Manual for Scientists: Oxford University Press; 2008.

[6] Halsey LG, Shepard EL, Hulston CJ, Venables MC, White CR, Jeukendrup AE, et al Acceleration versus heart rate for estimating energy expenditure and speed during locomotion in animals: Tests with an easy model species, *Homo sapiens* . Zoology. 2008;111(3):231–41. 10.1016/j.zool.2007.07.011 18375107

[7] Daley MA, Bramble DM, Carrier DR. Impact Loading and locomotor-respiratory coordination significantly influence breathing dynamics in running humans. PLoS ONE. 2013;8(8):e70752 10.1371/journal.pone.0070752 23950997 PMC3741319

[8] Bartholomew GA, Lighton J. Oxygen consumption during hover-feeding in free-ranging Anna hummingbirds. J Exp Biol. 1986;123(1):191–9. 3746193 10.1242/jeb.123.1.191

[9] Nagy KA. Field metabolic rate and food requirement scaling in mammals and birds. Ecol Monogr. 1987:112–28.

[10] Socha JJ, Lee W-K, Harrison JF, Waters JS, Fezzaa K, Westneat MW. Correlated patterns of tracheal compression and convective gas exchange in a carabid beetle. J Exp Biol. 2008;211(21):3409–20. 18931314 10.1242/jeb.019877

[11] Westneat MW, Betz O, Blob RW, Fezzaa K, Cooper WJ, Lee W-K. Tracheal respiration in insects visualized with synchrotron X-ray imaging. Science. 2003;299(5606):558–60. 12543973 10.1126/science.1078008

[12] Miller P. Respiration in the desert locust I. The control of ventilation. J Exp Biol. 1960;37(2):224–36.

[13] Gray EM, Bradley TJ. Evidence from mosquitoes suggests that cyclic gas exchange and discontinuous gas exchange are two manifestations of a single respiratory pattern. J Exp Biol. 2006;209(9):1603–11. 16621941 10.1242/jeb.02181

[14] Moon JK, Vohra FA, Valerio JO, Puyau MR, Butte NF. Closed-loop control of carbon dioxide concentration and pressure improves response of room respiration calorimeters. The Journal of Nutrition. 1995;125(2):220–8. 7861249 10.1093/jn/125.2.220

[15] Henning B, Löfgren R, Sjöström L. Chamber for indirect calorimetry with improved transient response. Medical and Biological Engineering and Computing. 1996;34(3):207–12. 8762827 10.1007/BF02520075

[16] Granato L, Brandes A, Bruni C, Greco AV, Mingrone G. VO2, VCO2, and RQ in a respiratory chamber: accurate estimation based on a new mathematical model using the Kalman-Bucy method. J Appl Physiol. 2004;96(3):1045–54. 14617529 10.1152/japplphysiol.00788.2003

[17] Tokuyama K, Ogata H, Katayose Y, Satoh M. Algorithm for transient response of whole body indirect calorimeter: deconvolution with a regularization parameter. J Appl Physiol. 2009;106(2):640–50. 10.1152/japplphysiol.90718.2008 19008487

[18] Bartholomew GA, Vleck D, Vleck CM. Instantaneous measurements of oxygen consumption during pre-flight warm-up and post-flight cooling in *sphingid* and *saturniid* moths. J Exp Biol. 1981;90(1):17–32.

[19] Williams T, Chambers J, May O, Henderson R, Rashotte M, Overton J. Concurrent reductions in blood pressure and metabolic rate during fasting in the unrestrained SHR. American Journal of Physiology-Regulatory, Integrative and Comparative Physiology. 2000;278(1):R255–R62. 10644647 10.1152/ajpregu.2000.278.1.R255

[20] Swallow JG, Garland T, Carter PA, Zhan W-Z, Sieck GC. Effects of voluntary activity and genetic selection on aerobic capacity in house mice (*Mus domesticus*). J Appl Physiol. 1998;84(1):69–76. 9451619 10.1152/jappl.1998.84.1.69

[21] Woakes AJ, and Butler P. J.. Swimming and diving in tufted ducks, Aythya fuligula, with particular reference to heart rate and gas exchange. J Exp Biol. 1983;107(1):311–29.

[22] Parkes R, Halsey L. G, Woakes A. J., Holder R. L., & Butler P. J.. Oxygen uptake during post dive recovery in a diving bird Aythya fuligula: implications for optimal foraging models. J Exp Biol. 2002;205(24):3945–54. 12432016 10.1242/jeb.205.24.3945

[23] Sun M, Reed G, Hill J. Modification of a whole room indirect calorimeter for measurement of rapid changes in energy expenditure. J Appl Physiol. 1994;76(6):2686–91. 7928901 10.1152/jappl.1994.76.6.2686

[24] Brychta RJ, Rothney MP, Skarulis MC, Chen KY, editors. Optimizing energy expenditure detection in human metabolic chambers. Engineering in Medicine and Biology Society, 2009 EMBC 2009 Annual International Conference of the IEEE; 2009: IEEE. 10.1109/IEMBS.2009.5333121 PMC6467262 19964185

[25] Frappell P, Blevin H, Baudinette R. Understanding respirometry chambers: what goes in must come out. J Theor Biol. 1989;138(4):479–94. 2593683 10.1016/s0022-5193(89)80046-3

[26] Lighton JR. ‘Instantaneous’ metabolic measurement J Exp Biol. 2012;215(10):1605–6. 22539726 10.1242/jeb.068288

[27] Chartrand R. Numerical differentiation of noisy, nonsmooth data. ISRN Applied Mathematics. 2011;2011.

[28] Porat B. A course in digital signal processing: Wiley New York; 1997.

[29] Hamilton JD. Time series analysis: Princeton University Press Princeton; 1994.

[30] Contreras H, Bradley T. Transitions in insect respiratory patterns are controlled by changes in metabolic rate. J Insect Physiol. 2010;56(5):522–8. 10.1016/j.jinsphys.2009.05.018 19523955

[31] Pendar H. Input estimation of linear time-invariant systems using GZT method. Available: https://github.com/TheSochaLab/GZT-method-for-data-recovery 2015.

[32] Mault JR, Pearce Jr EM. Indirect calorimeter for medical applications. U.S. Patent No. 6,629,934). 2003.

[33] Veldhuis JD, Carlson ML, Johnson ML. The pituitary gland secretes in bursts: appraising the nature of glandular secretory impulses by simultaneous multiple-parameter deconvolution of plasma hormone concentrations. Proceedings of the National Academy of Sciences. 1987;84(21):7686–90. 10.1073/pnas.84.21.7686 PMC299365 2823271

[34] Giustina A, Veldhuis JD. Pathophysiology of the neuroregulation of growth hormone secretion in experimental animals and the human 1. Endocrine Reviews. 1998;19(6):717–97. 9861545 10.1210/edrv.19.6.0353

[35] Vicini P, Sparacino G, Caumo A, Cobelli C. Estimation of endogenous glucose production after a glucose perturbation by nonparametric stochastic deconvolution. Computer Methods and Programs in Biomedicine. 1997;52(3):147–56. 9051338 10.1016/s0169-2607(96)01784-1

[36] Sparacino G, Cobelli C. A stochastic deconvolution method to reconstruct insulin secretion rate after a glucose stimulus. Biomedical Engineering, IEEE Transactions on. 1996;43(5):512–29. 10.1109/10.488799 8849464

[37] De Nicolao G, Liberati D, Sartorio A. Deconvolution of infrequently sampled data for the estimation of growth hormone secretion. IEEE Transactions on Biomedical Engineering. 1995;42(7):678–87. 7542624 10.1109/10.391166

[38] De Nicolao G, Sparacino G, Cobelli C. Nonparametric input estimation in physiological systems: problems, methods, and case studies. Automatica. 1997;33(5):851–70.

[39] Bartholomew GA, Barnhart MC. Tracheal gases, respiratory gas exchange, body temperature and flight in some tropical cicadas. J Exp Biol. 1984;111(1):131–44.

[40] Ortigues I, Dussap C-G, Anglaret Y. Comparison of various methods of calculating the instantaneous respiratory gaseous exchanges from discrete measurements in respiration chambers. J Theor Biol. 1997;185(4):489–501.

[41] Tartes U, Vanatoa A, Kuusik A. The insect abdomen—a heartbeat manager in insects? Comp Biochem Physiol, A: Comp Physio. 2002;133(3):611–23. 10.1016/s1095-6433(02)00173-3 12443919

[42] Sláma K. A new look at insect respiration. Biol Bull. 1988;175(2):289–300.

