# Correction: Estimation of Instantaneous Gas Exchange in Flow-Through Respirometry Systems: A Modern Revision of Bartholomew's Z-Transform Method

**DOI:** 10.1371/journal.pone.0142959

**Published:** 2015-11-16

**Authors:** The PLOS ONE Staff

There is an error in the first sentence of the sixth paragraph of the ZT Method section of the Methods. The publisher apologizes for this error. The correct sentence is: Equation 4 directly determines the instantaneous gas exchange rate (*u*) from the recorded data:

$$u(t)=c(t)+\frac{c(t+dt)-c(t)}{Z}$$

where $Z=\frac{F}{V}dt$, and it requires high sampling rate of the outlet gas.

In the Generalized ZT (GZT) method section of the Methods, there is an error in the 15^th^ equation. Please view the complete, correct equation here:

$$C=\left(\begin{array}{c} C_{0}\\ C_{1}\\ \vdots \\ C_{n} \end{array}\right),\;C_{j}=\left(c(j),\cdots ,c(j+N)\right)$$

There is an error in reference 21. The correct reference is: Woakes AJ, and Butler P. J. Swimming and diving in tufted ducks, *Aythya fuligula*, with particular reference to heart rate and gas exchange. J Exp Biol. 1983;107(1):311–29.

There are errors in Tables 1–3. Please see the correct Tables 1–3 here.

**Table 1:** Parameters of the impulse responses. Experimentally-determined impulse responses of three respirometry chambers in five different flow rates were modeled in four ways. In the first three models, αt^m^e^-βt^ has been fitted to the data. Here m, α, and β are constant parameters that are needed to fit this curve (αt^m^e^-βt^) to the experimental data (see text for details). In the first one, *m* is considered as a real number. In the second, one we restricted the *m* to be an integer and then found the parameters. In the third one, we decreased *m* to the lowest integer number without having more than 10% ITAE ($\int_{0}^{\infty }\;|e|dt$). In the last one, we forced *m* to be zero in order to recover the ZT model.

		Best fit curve			EZT method						ZT method	
					Best integer *m*			Small *m*			(*m = 0*)	
V(mL)	F(mL/min)	*m*	*β* (1/s)	*δ* (s)	*m*	*β*	*δ*	*m*	*β*	*δ*	*β*	*δ*
28	125	1.65	0.188	12.75	2	0.206	12.05	1	0.15	14.16	0.066	16.62
28	250	0.97	0.246	5.87	1	0.249	5.82	1	0.249	5.82	0.1094	7.204
28	500	1.16	0.49	4.08	1	0.459	4.19	1	0.459	4.19	0.2018	5.025
28	1250	2.85	2.089	1.5	3	2.142	1.47	2	1.761	1.68	0.5657	2.236
28	2500	14.2	6.58	0.29	14	6.53	0.29	3	3.045	0.79	0.8091	1.338
125	125	0.86	0.054	18.9	1	0.057	18.09	1	0.057	18.09	0.2441	23.95
125	250	1.83	0.178	10.58	2	0.185	10.2	1	0.134	12.51	0.0575	15.15
125	500	1.12	0.246	6.27	1	0.234	6.44	1	0.234	6.44	0.1011	8.0
125	1250	0.35	0.291	2.46	0	0.189	2.74	0	0.189	2.74	0.1892	2.74
125	2500	0.67	0.592	1.42	1	0.699	1.26	1	0.699	1.26	0.3057	1.77
600	125	0.026	0.005	29.36	0	0.0035	29.75	0	0.0035	29.75	0.0035	29.75
600	250	0.048	0.011	19.91	0	0.0102	20.46	0	0.0102	20.46	0.0102	20.46
600	500	0.144	0.027	13.83	0	0.0217	14.65	0	0.0217	14.65	0.0217	14.65
600	1250	0.206	0.042	14.36	0	0.0313	15.33	0	0.0313	15.33	0.0313	15.33
600	2500	0.204	0.131	3.03	0	0.0969	3.26	0	0.0969	3.26	0.0968	3.26

**Table 2:** Estimated parameters of ZT and EZT methods using Eqs 2 and 11 and from experimental data.

Method	F(mL/min)	Parameterestimation method	a_1_	a_2_
ZT	250	Equation 2	6.72	-
ZT	250	Experiment	12.2	-
ZT	500	Equation 2	3.36	-
ZT	500	Experiment	5.3	-
EZT	250	Equation 12	8.03	16.13
EZT	250	Experiment	12.3	41.2
EZT	500	Equation 12	4.36	4.75
EZT	500	Experiment	5.2	5.1

**Table 3:** Normalized ITAE to the area of the input signal of the recovered signals in different frequencies.

		Normalized ITAE				
Method	F(mL/mi)	0.1 Hz	0.167Hz	0.25 Hz	0.5 Hz	1 Hz
ZT	250	1.1767	1.1667	1.1683	1.5375	1.6032
EZT	250	0.7577	0.6889	0.8073	1.2268	1.3170
GZT	250	0.3269	0.4096	0.4757	0.7862	1.2056
ZT	500	0.4500	0.7904	0.9496	1.1264	1.4902
EZT	500	0.3724	0.5400	0.9004	1.2114	1.4116
GZT	500	0.2699	0.4855	0.5010	0.7204	1.3549

## References

[1] Pendar H, Socha JJ (2015) Estimation of Instantaneous Gas Exchange in Flow-Through Respirometry Systems: A Modern Revision of Bartholomew's Z-Transform Method. PLoS ONE 10(10): e0139508 doi:10.1371/journal.pone.0139508 26466361 10.1371/journal.pone.0139508 PMC4605654

