# Supplementary material for: Estimation of Instantaneous Gas Exchange in Flow-Through Respirometry Systems: A Modern Revision of Bartholomew's Z-Transform Method
Source: PLoS One. 2015 Oct 14;10(10):e0139508. doi: 10.1371/journal.pone.0139508 (PMC4605654; doi:10.1371/journal.pone.0139508)
Supplement: S1 File — (PDF) [file pone.0139508.s001.pdf]

Appendix-A – Reformulation of the Z-transform method

McLean & Watts [1] and Bartholomew and et. al. [2] used the following method, also known as the Z-transform method, to estimate instantaneous oxygen consumption ($\dot{V}_{O_2}$) from the fractional concentration of oxygen (F_E) in the excurrent air stream from the respirometry chamber. This method assumes that after any changes in $\dot{V}_{O_2}$ in an open-flow respirometry system, F_E changes exponentially to a new steady-state equilibrium value, $F_{E_{eq}}$. To accurately estimate $\dot{V}_{O_2}$, we require not only the current F_E, but also the equilibrium value of the fractional concentration ($F_{E_{eq}}$) that would eventually be reached if there were no further change in $\dot{V}_{O_2}$. For any system with exponential washout behavior, such as a flow-through respirometry system, the rate of approach to the equilibrium point is constant and it can be determined as follows:

$$Z = \frac{F_E(k+1) - F_E(k)}{F_{E_{eq}} - F_E(k)} \qquad \text{(S1-A1)}$$

where

$$Z = 1 - e^{-\frac{F}{V}T} \qquad \text{(S1-A2)}$$

and T is the measurement time interval. $F_{E_{eq}}$ can be determined by combining these two equations:

$$F_{E_{eq}} = \frac{F_E(k+1) - F_E(k)}{1 - e^{-\frac{F}{V}T}} + F_E(k) \qquad \text{(S1-A3)}$$

Therefore, $F_{E_{eq}}$ can be calculated by two consequent measurement of F_E.

The instantaneous change of oxygen in the chamber can be determined by using $F_{E_{eq}}$ as follows [3]:

$$\dot{V}_{O_2} = F\frac{F_{I_{O2}} - F_{E_{eq}}}{1 - F_{I_{O2}}} \tag{S1-A4}$$

where F is the flow rate and $F_{I_{O2}}$ is the fractional concentration of oxygen in the inlet air flow. By defining $u(k) = \frac{\dot{V}_{O_2}}{F}$ and $c(k) = \frac{F_{I_{O2}} - F_E(k)}{1 - F_{I_{O2}}}$ (the change in the concentration of the gas), Equation S1-A4 changes to:

$$u(k) = \frac{1}{Z}c(k+1) - \frac{1-Z}{Z}c(k) \tag{S1-A5}$$

which is Equation 1 in the manuscript. As long as the washout is exponential, the same equations are valid for CO_2 or any other gases.

Appendix-B – Discretizing a differential equation

The dynamical equations of any linear system has the following state-space format [4]:

$$\dot{\mathbf{X}}(t) = \mathbf{A}\mathbf{X}(t) + \mathbf{B}u(t) \qquad \text{(S1-B1)}$$

and can be transformed to a difference equation or discrete model, following [4]:

$$\mathbf{X}(k+1) = \mathbf{A}_d\mathbf{X}(k) + \mathbf{B}_d u(k) \qquad \text{(S1-B2)}$$

where:

$$\mathbf{A}_d = e^{\mathbf{A}T} \qquad \text{(S1-B3)}$$

$$\mathbf{B}_d = \left(\int_0^T e^{\mathbf{A}\tau}\, d\tau\right)\mathbf{B} = \mathbf{A}^{-1}(\mathbf{A}_d - \mathbf{I})\mathbf{B} \qquad \text{(S1-B4)}$$

Therefore, the discrete form of Equation 4 ($u(t) = c(t) + \frac{1}{F/V}\dot{c}(t)$) is:

$$u(k) = \frac{F}{1 - e^{-\frac{F}{V}T}}\left(c(k+1) - e^{-\frac{F}{V}T}c(k)\right) \qquad \text{(S1-B5)}$$

which is equivalent to Equations 1 and 6.

Appendix-C – Experimental determination of the impulse response

The impulse response, *h(t)*, can be experimentally determined by injecting a short pulse of CO_2 into the chamber close to where the animal emits CO_2. To obtain the impulse response precisely, two aspects of methodology should be considered carefully. First, the duration of the CO_2 pulse should be very small in comparison to the duration of the impulse response. The exact duration of the impulse is not critical, but it should be very small (based on experience, it should be less than 0.5% of the impulse response duration). If the respirometry chamber is relatively large or the flow rate is relatively small, the pulse duration can be long. In those cases, it is possible to find the impulse response by manually blowing into the chamber. In a system with fast dynamics, i.e., one with a high flow rate and/or a small chamber, a high-speed valve is required to precisely control the duration of the injection. Second, the injection should not significantly affect the flow rate. If it changes the flow rate, then the system is no longer an LTI system. This means that the volumetric injection rate must also be negligible compared to volume flow rate the inlet airflow.

If these assumptions are met, the actual concentration and pattern of the gas injection used by the experimenter does not matter, because the measured impulse response will be not be affected (S1 Fig. 1). Here, we explain why this is true. Transforming the input to an output is an area-conservative process because all injected CO_2 molecules eventually pass through the IR chamber of the sensor. Therefore, by normalizing the output signal by dividing by its area, the output of the gas analyzer becomes independent of the shape of the input signal. To demonstrate this concept, a convolution of different short inputs with an impulse response has been computed in a simulation using Equation 7. The normalized outputs are very close to the true impulse response (S1 Fig. 1), even if the outputs are very different. We also tested this experimentally, by injecting different pulses of CO_2 with different durations into the 28 *mL* chamber, using with different pulse sizes (with flow rate of 125 mL/min). The results (S1 Figs. 1B and 1C) demonstrate the independence of the result and the shape of the short pulses. A Picospritzer microinjector (Picospritzer III, Parker Hannifin, Precision Fluidics Division, NH, US) was used to control the duration of the pulses. With the microinjector, the duration of the pulse can be

precisely controlled, but the flow rate depends on the pressure of the upstream flow. To provide an extreme condition, we changed the pressure of the CO_2 upstream (before the injection valve) for each pulse. The outputs of the respirometry system are different for each pulse, but as shown in S1 Fig. 1C, the normalized outputs that represent the impulse response are indeed identical.

This study showed that the expression $\alpha t^m e^{-\beta t}$ adequately describes the impulse responses of small respirometry chambers. In this model, the parameter α is not independent from the other two parameters. Because the area of the impulse response is one, α can be determined to normalize the impulse response as follows:

$$\alpha = \frac{1}{\int_0^\infty t^m e^{-\beta t}\, dt} = \frac{\beta^{m+1}}{m!} \qquad \text{(S1-C1)}$$

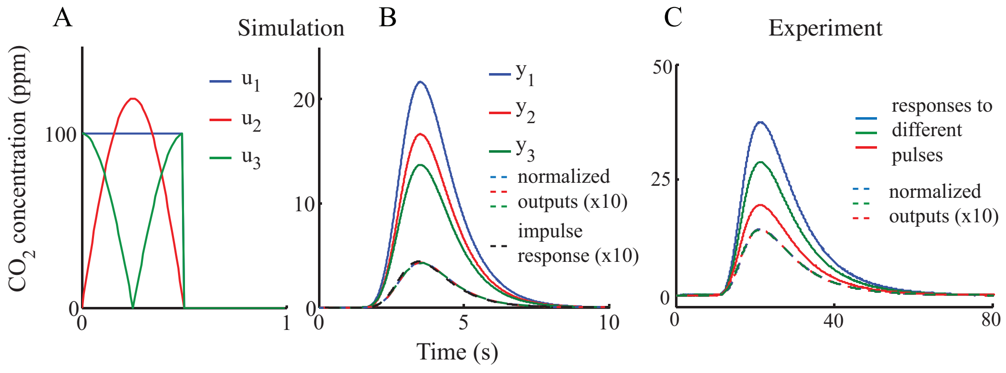

S1 Fig. 1: The impulse response can be determined by injecting a short input. If the duration of the input is small compared to the duration of the impulse response, the normalized output signal (the impulse response) will be independent of the shape of the input signal. (A) and (B) show the different input signals and their corresponding outputs in a simulation for a given impulse response. The normalized outputs are very close to the given impulse response. The determined impulse responses for a setup (V = 28 *mL*, F = 125 *mL/min*) with different inputs are indistinguishable (C).

Appendix-D – Derivation of the governing equation of the system using the impulse response

The Laplace-transformed form of the convolution equation of Equation 7 is:

$$C(s) = H(s)U(s) \quad \text{(S1-D1)}$$

$$U(s) = \frac{C(s)}{H(s)} \quad \text{(S1-D2)}$$

where *C(s)*, *H(s)*, and *U(s)* are the Laplace transforms of *c(t)*, *h(t)*, and *u(t),* respectively. The Laplace transform of the impulse response $h(t) = \alpha t^m e^{-\beta t}$ is:

$$H(s) = \frac{\alpha m!}{(s+\beta)^{m+1}} \quad \text{(S1-D3)}$$

Plugging S1-D3 into S1-D2 yields:

$$U(s) = \frac{1}{\alpha m!}(s+\beta)^{m+1} C(s) \quad \text{(S1-D4)}$$

The inverse Laplace transform of the above equation is:

$$u(t) = \frac{1}{\alpha m!} e^{-\beta t} \frac{d^{m+1}}{dt^{m+1}}\left(e^{\beta t} c(t)\right) \quad \text{(S1-D5)}$$

Appendix-E – Robust differentiation of noisy data

Several methods can be used to differentiate noisy data and regulate the detrimental amplification of noise [5-11]. Here we used the low-noise Lanczos differentiator method, in which at each point, several neighboring points are used to calculate the derivative. For instance by using seven points, the derivative of a function *f(x)* at x^* will be:

$$f(x^*) = \frac{126(f_1 - f_{-1}) + 193(f_2 - f_{-2}) + 142(f_3 - f_{-3}) - 86(f_4 - f_{-4})}{1188\Delta T} \quad \text{(S1-E1)}$$

For derivations with a different number of points, see [10].

Appendix-F – Woakes' method

After Bartholomew's paper introduced the Z-transform in 1981 [2], Woakes and Butler published another method to recover the instantaneous gas exchange signal in flow-through respirometry systems in 1983 [12]. Although they derived their equations with different approaches, here we show that these two methods are actually identical. It is possible that Woakes and Butler were unaware of the Z-transform when they developed their version of the method.

In the appendix of their paper [12], Woakes and Butler provide the following equation for the volume of the produced CO_2 or consumed O_2:

$$V_{gas} = (C_2 - C_1)V + \frac{(C_1 + C_2)}{2}(t_2 - t_1)\dot{Q} \tag{S1-F1}$$

where t_1 and t_2 are sampling times, C_1 and C_2 are the fractional concentrations of the gas in the outlet in respective times, V is the chamber volume, and $\dot{Q}$ is the flow rate (which is called F in our manuscript). In this method, it is assumed that the fractional concentration of the gas changes almost linearly from C_1 to C_2 during this interval. By dividing both sides of the equation by $(t_2 - t_1)\dot{Q}$, which is the total inlet gas from t_1 to t_2, we can find the instantaneous flow rate of the produced CO_2 or consumed O_2 during this interval. This quantity is named u in the main text:

$$u = \frac{V_{gas}}{(t_2 - t_1)\dot{Q}} = \frac{(C_2 - C_1)}{(t_2 - t_1)}\frac{V}{\dot{Q}} + \frac{(C_1 + C_2)}{2} \tag{S1-F2}$$

Note that $\frac{(C_1 + C_2)}{2}$ and $\frac{(C_2 - C_1)}{(t_2 - t_1)}$ are the fractional gas concentration and the time derivative of it at $t = \frac{(t_2 + t_1)}{2}$, respectively. Replacing these quantities with $c(t)$ and $\dot{c}(t)$ in equation S1-F2 results in equation 4, which is the Z-transform or Bartholomew equation.

Appendix-G – MATLAB code

A MATLAB code with sample experimental data is provided [13] (S2 file) to perform the GZT method on experimental data. Before using the method in an experiment, the calibration coefficients must be determined. CO_2 gas (or any other measurable gases) with an arbitrary pattern (*u*(t), S1 Fig. 2) should be injected into the respirometry chamber and the concentration of this gas in the outlet should be recorded (*c*(t), S1 Fig. 2). For this code, both signals should be saved in a text file with the name 'CalibrationData.txt', which contains three columns of data: time, the input signal *u*(t), and the output signal *c*(t). After running the code and selecting the calibration option, the code uses Equation 14 to determine the calibration coefficients and saves them in the 'Parameters_a.txt' file. After determining the calibration coefficients, the experimenter can now recover the instantaneous gas exchange data from their raw gas exchange data. After collecting raw gas exchange data from the subject, the recorded data should be saved in a text file with the name of 'Data.txt', which should contain two columns: time, and concentration of the gas of interest in the outlet (Fig. 7). After running the code and choosing the signal recovery option, the code uses the raw data and the calibration coefficients the recover the instantaneous gas exchange signal (Fig. 7).

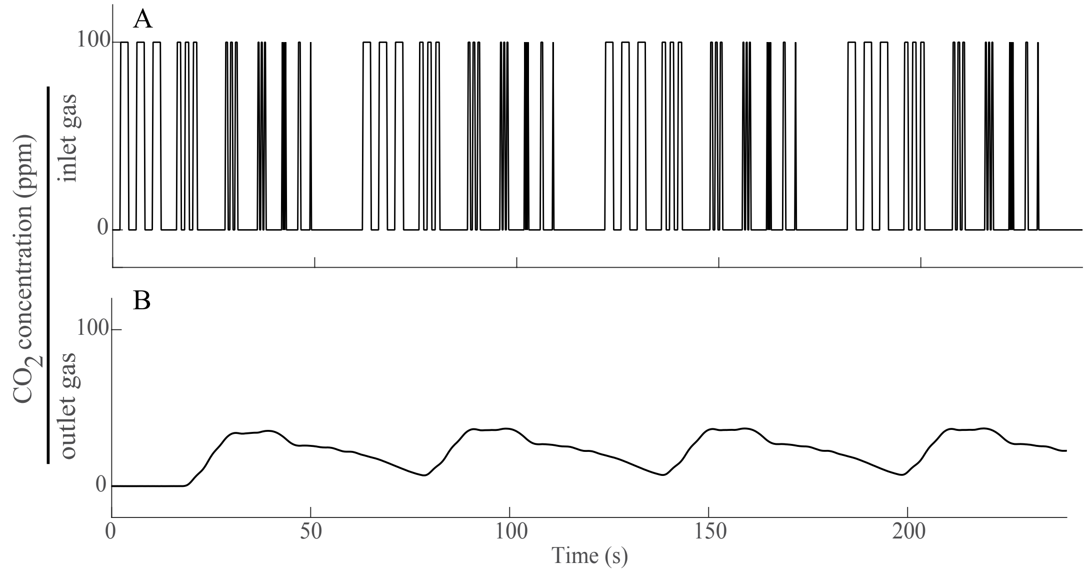

S1 Fig. 2: A known concentration of CO_2 is infused into the chamber (A) and the CO_2 concentration in the outlet of the respirometry chamber (B) is recorded for use in calibration.

References

1. McLean J, Watts P (1976) Analytical refinements in animal calorimetry. *Journal of Applied Physiology* 40: 827-831.
2. Bartholomew GA, Vleck D, Vleck CM (1981) Instantaneous measurements of oxygen consumption during pre-flight warm-up and post-flight cooling in *sphingid* and *saturniid* moths. *Journal of Experimental Biology* 90: 17-32.
3. Withers PC (1977) Measurement of VO2, VCO2, and evaporative water loss with a flow-through mask. *Journal of Applied Physiology* 42: 120-123.
4. Franklin GF, Workman ML, Powell D (1997) Digital control of dynamic systems: Addison-Wesley Longman Publishing Co., Inc.
5. Savitzky A, Golay MJ (1964) Smoothing and differentiation of data by simplified least squares procedures. *Analytical Chemistry* 36: 1627-1639.
6. Ahnert K, Abel M (2007) Numerical differentiation of experimental data: local versus global methods. *Computer Physics Communications* 177: 764-774.
7. Wei T, Li M (2006) High order numerical derivatives for one-dimensional scattered noisy data. Applied Mathematics and Computation 175: 1744-1759.
8. Cullum J (1971) Numerical differentiation and regularization. *SIAM Journal on Numerical Analysis* 8: 254-265.
9. Chartrand R (2011) Numerical differentiation of noisy, nonsmooth data. *ISRN Applied Mathematics* 2011.
10. Holoborodko P (2008) Smooth noise robust differentiators, http://www.holoborodko.com/pavel/.
11. Walker JA (1998) Estimating velocities and accelerations of animal locomotion: a simulation experiment comparing numerical differentiation algorithms. *The Journal of Experimental Biology* 201: 981-995.
12. Woakes AJ, and P. J. Butler. (1983) Swimming and diving in tufted ducks, Aythya fuligula, with particular reference to heart rate and gas exchange. *Journal of Experimental Biology* 107: 311-329.
13. Pendar H (2015) Input estimation of linear time-invariant systems using the GZT method, https://github.com/TheSochaLab/GZT-method-for-data-recovery.
